# Increased risk of ACL revision with non-surgical treatment of a concomitant medial collateral ligament injury: a study on 19,457 patients from the Swedish National Knee Ligament Registry

**DOI:** 10.1007/s00167-018-5237-3

**Published:** 2018-10-29

**Authors:** Eleonor Svantesson, Eric Hamrin Senorski, Eduard Alentorn-Geli, Olof Westin, David Sundemo, Alberto Grassi, Svemir Čustović, Kristian Samuelsson

**Affiliations:** 1 Department of Orthopaedics, Institute of Clinical Sciences, The Sahlgrenska Academy, University of Gothenburg, Gothenburg, Sweden; 2 Department of Health and Rehabilitation, Institute of Neuroscience and Physiology, The Sahlgrenska Academy, University of Gothenburg, Gothenburg, Sweden; 3 Fundación García Cugat, Barcelona, Spain; 4 Artroscopia GC, Hospital Quirón, Barcelona, Spain; 5 Mutualidad Catalana de Futbolistas, Federación Catalana de Fútbol, Barcelona, Spain; 6 Department of Orthopaedics, Sahlgrenska University Hospital, Mölndal, Sweden; 7 2nd Orthopaedic and Traumatologic Clinic, IRCCS Rizzoli Orthopaedic Institute, Bologna, Italy; 8 Department of Biomedic and Neuromotor Sciences DIBINEM, University of Bologna, Bologna, Italy; 9 Clinic for Orthopaedics and Traumatology, University Clinical Centre Tuzla, Tuzla, Bosnia and Herzegovina

**Keywords:** Anterior cruciate ligament (ACL), Medial collateral ligament (MCL), Lateral collateral ligament (LCL), Reconstruction, Revision, Registry

## Abstract

**Purpose:**

To determine how concomitant medial collateral ligament (MCL) and lateral collateral ligament (LCL) injuries affect outcome after anterior cruciate ligament (ACL) reconstruction.

**Methods:**

Patients aged > 15 years who were registered in the Swedish National Knee Ligament Registry for primary ACL reconstruction between 2005 and 2016 were eligible for inclusion. Patients with a concomitant MCL or LCL injury were stratified according to collateral ligament treatment (non-surgical, repair or reconstruction), and one isolated ACL reconstruction group was created. The outcomes were ACL revision and the 2-year Knee Injury and Osteoarthritis Outcome Score (KOOS), which were analyzed using univariable and multivariable Cox regression and an analysis of covariance, respectively.

**Results:**

A total of 19,457 patients (mean age 27.9 years, 59.4% males) met the inclusion criteria. An isolated ACL reconstruction implied a lower risk of ACL revision compared with presence of a non-surgically treated MCL injury (HR = 0.61 [95% CI 0.41–0.89], *p* = 0.0097) but not compared with MCL repair or reconstruction. A concomitant LCL injury did not impact the risk of ACL revision. Patients with a concomitant MCL or LCL injury reported inferior 2-year KOOS compared with isolated ACL reconstruction. The largest difference was found in the sports and recreation subscale across all groups, with MCL reconstruction resulting in the maximum difference (14.1 points [95% CI 4.3–23.9], *p* = 0.005).

**Conclusion:**

Non-surgical treatment of a concomitant MCL injury in the setting of an ACL reconstruction may increase the risk of ACL revision. However, surgical treatment of the MCL injury was associated with a worse two-year patient-reported knee function. A concomitant LCL injury does not impact the risk of ACL revision compared with an isolated ACL reconstruction.

**Level of evidence:**

Cohort study, Level III.

## Introduction

The medial collateral ligament (MCL) and the lateral collateral ligament (LCL) of the knee joint are primary restraints to valgus and varus forces, respectively, and do also counteract tibial rotation [11, 30]. The shared stabilizing properties of the collateral ligaments and the anterior cruciate ligament (ACL) mean that the risk of combined injuries to these structures is high. In spite of this, consensus has not yet been reached in terms of the optimal treatment for combined injuries and there is sparse evidence relating to the way the presence and treatment of concomitant collateral ligament injuries affect the outcome after primary ACL reconstruction.

Successful outcomes after non-surgical treatment of isolated MCL injuries [17, 18, 25, 26, 29] have led many surgeons to advocate non-surgical treatment for the MCL also in combined ACL and MCL injuries, with early or delayed ACL reconstruction [12, 13, 16, 27, 28, 32, 34, 39]. However, MCL deficiency has been reported to increase the forces on the ACL graft [1, 43] and surgical intervention to re-establish the stabilizing properties of the MCL may benefit the healing of both the ACL graft and the MCL [37]. Several techniques for the surgical treatment of the MCL, involving both suture repair and ligament reconstruction, have been described and reported to produce successful outcomes [2, 4, 5, 9, 21, 26, 42].

Clinical studies of combined ACL and LCL injuries have so far been limited in the literature. Nonetheless, the role of the LCL in preventing the anterolateral rotatory instability of the ACL-injured knee has been established [40] and addressing lateral deficiency is regarded as crucial to restore kinematics in the ACL-reconstructed knee [41]. Instability caused by insufficiency of the posterolateral structures of the knee joint increases the risk of graft failure in a simultaneous ACL reconstruction [3, 10, 33], thereby underlining the importance of a thorough assessment and treatment of involved structures, including the LCL.

The purpose of this national registry study was to determine how presence and treatment of concomitant MCL or LCL injuries affect the risk of ACL revision and patient-reported knee function after primary ACL reconstruction compared with isolated ACL reconstruction. It was hypothesized that non-surgical treatment of a concomitant collateral ligament injury at the time of ACL reconstruction would increase the risk of ACL revision and result in inferior patient-reported knee function compared with surgical treatment.

## Materials and methods

Data from the Swedish National Knee Ligament Registry (SNKLR) were collected prospectively between January 1, 2005 through December 31, 2016. The inclusion criteria for the study were primary single-bundle ACL reconstruction with hamstring tendon (HT) or patella tendon (PT) autografts and age > 15 years. Patients fulfilling any of the following criteria were excluded from analysis; the first entry in the SNKLR was ACL revision, prior contralateral ACL reconstruction, a concomitant fracture/nerve injury/vascular injury or posterior cruciate ligament (PCL) injury, double-bundle ACL reconstruction, a graft type for ACL reconstruction other than PT or HT, an unregistered HT graft diameter, combined MCL and LCL injuries or contralateral MCL or LCL surgery.

### The Swedish National Knee Ligament Registry

The SNKLR is a nationwide registry established in January 2005 and it comprises over 90% of the ACL reconstructions performed annually in Sweden [6]. The SNKLR is divided into two separate parts, one surgeon-reported and one patient-reported. The surgeon enters information regarding identified knee joint injuries, such as ligament, meniscal and cartilage injuries, and reports details of the surgical treatment. Any additional surgeries, such as revision or contralateral ACL reconstruction, are entered as separate events in the database and linked to the patient’s primary ACL reconstruction. The patient-reported part includes demographic data and prospectively collected patient-reported outcome in terms of the European Quality of Life-5 Dimensions (EQ-5D) and the Knee Injury and Osteoarthritis Outcome Score (KOOS) [31]. The patient-reported outcomes are administered preoperatively and at 1-, 2-, 5- and 10-year follow-ups.

### Study groups

All the patients in the study cohort underwent primary ACL reconstruction and were stratified by the presence and treatment of a concomitant MCL or LCL injury. The stratification yielded a total of seven separate study groups for analysis. The isolated ACL group was defined as patients undergoing primary ACL reconstruction without concomitant MCL or LCL injury. Patients with a concomitant MCL or LCL injury were identified in the surgeon-reported part of the SNKLR and three groups for the MCL- and the LCL-injured patients, respectively, were created as follows: non-surgically-treated, suture repair and reconstruction. A suture repair of the MCL or LCL is directly reported in the SNKLR and it was therefore identified accordingly in the database. However, the data entry design in the SNKLR does not enable the reporting of a specific method for the surgical reconstruction of a collateral ligament. The surgical reconstruction of an MCL or LCL injury is reported, as the surgeon enters a combination of (1) the presence of a concomitant MCL or LCL injury and (2) the use of a graft in relation to the MCL or LCL injury. Patients with an MCL or LCL injury who did not fulfill the criteria for either suture repair or reconstruction formed the non-surgically-treated MCL and LCL groups.

### Outcome

The primary outcomes were ACL revision and the KOOS at the 2-year follow-up. An ACL revision was defined as the patient undergoing an ACL revision during the follow-up period, which started at the date of the patient’s index ACL reconstruction and ended on December 31, 2016 or at the date of the ACL revision. The KOOS is a patient-reported outcome measurement comprising five subscales—knee-related symptoms, pain, activities of daily living (ADL), function in sports and recreation and knee-related quality of life (QoL). Each subscale yields a possible score of 0–100, where a score of zero represents the worst possible knee problems and a score of 100 indicates no knee problems [31]. The KOOS_4_ is a modified version of the KOOS, where the ADL subscale is excluded due to a potential ceiling effect since most active and young patients do not experience difficulties in ADL. The KOOS_4_ is therefore an average score of the remaining four subscales, similarly ranging from 0 to 100 [8].

Participation in the SNKLR is voluntary for both patients and surgeons. All patients received written information regarding the participation. Ethical approval was obtained from the Regional Ethical Review Board in Stockholm (registration number: 2011/337-31/3).

### Statistical analysis

All statistical analyses were performed using Statistical Analysis System software (SAS/STAT, version 14.2, 2016; SAS Institute Inc., Cary, North Carolina, USA). Patient demographics and baseline patient-reported outcomes were presented as numbers and percentages for categorical variables and continuous variables as the mean and standard deviation (SD) across all study groups. For comparisons between two groups (with the isolated ACL group as reference), Fisher’s exact test was used for dichotomous variables, the Chi square test for non-ordered categorical variables and the Mann–Whitney *U* test for continuous variables. For comparisons between non-ordered groups, the Chi square test was used for non-ordered categorical variables (such as crude revision rate) and the Kruskal–Wallis test was used for continuous variables (such as the KOOS). To compare the 2-year KOOS between each group with a concomitant MCL or LCL injury and the isolated ACL reconstruction group (reference), an analysis of covariance (ANCOVA) was used. The ANCOVA was adjusted for possible confounding differences between the groups in demographic data known to affect the KOOS, i.e., age at surgery, patient sex and cartilage injury. Univariable and adjusted multivariable Cox regression analyses were performed to determine the proportional hazard ratio of ACL reconstruction survival or ACL revision between the isolated ACL group and the other groups. The Cox proportional hazard model analyzes time to ACL revision. Patients who underwent contralateral ACL reconstruction during follow-up were censored in the analyses. The Cox regression models were adjusted for patient sex, graft diameter and any additionally identified baseline confounders, defined as variables differing significantly between treatment groups, if these variables were also shown to correlate with both the predictor and the outcome of the regression model when tested. The proportional hazards assumptions were fulfilled and were investigated by visually reviewing the log(− log(Survival)) versus log(time) curves and by introducing an interaction term between each predictor and the logarithm of time in the study into the model. The results of the Cox regression analyses were reported as hazard ratios (HR), 95% confidence intervals (CI) and *p* values. All statistical tests were two-sided and conducted at the 5% significance level.

## Results

A total of 19,457 patients (mean age 27.9 years, 59.4% males) met the inclusion criteria, of which 18,490 patients formed the reference group of isolated ACL reconstruction in absence of a collateral ligament injury. Patients with a concomitant MCL injury were stratified by treatment; non-surgically-treated MCL (*n* = 657), MCL suture repair (*n* = 52) and MCL reconstruction (*n* = 84). The same stratification was made for patients with a concomitant LCL injury; non-surgically-treated LCL injury (*n* = 124), LCL suture repair (*n* = 15) and LCL reconstruction (*n* = 35). All patient baseline data and the paired comparative analysis between each MCL and LCL group and the isolated ACL reconstruction group are presented in Table 1.

**Table 1:** Baseline demographic data for study groups

Variable	Total (*n* = 19,457)	Isolated ACL (*n* = 18,490)	ACL + non-surgically treated MCL (*n* = 657)	ACL + MCL suture repair (*n* = 52)	ACL + MCL reconstruction (*n* = 84)	ACL + non-surgically treated LCL (*n* = 124)	ACL + LCL suture repair (*n* = 15)	ACL + LCL reconstruction (*n* = 35)
Age at surgery	27.9 (9.8)	27.8 (9.8)	30.7 (11.2)***	32.6 (12.2)**	30.3 (11.4)	28.5 (10.7)	25.7 (9.3)	27.6 (9.5)
Patient sex								
Male	11,548 (59.4%)	10,964 (59.3%)	367 (55.9%)	33 (63.5%)	59 (70.2%)	90 (72.6%)**	11 (73.3%)	24 (68.6%)
Cartilage injury								
Yes	5478 (28.2%)	5147 (27.8%)	212 (32.3%)*	15 (28.8%)	40 (47.6%)***	44 (35.5%)	1 (6.7%)	19 (54.3%)**
Femoral ACL fixation								
Endo-/Retrobutton/Tightrope	13,267 (68.5%)	12,616 (68.5%)	440 (67.8%)	41 (82.0%)	45 (54.2%)	89 (71.8%)	11 (73.3%)	25 (71.4%)
Rigid-/Transfix	2875 (14.8%)	2754 (15.0%)	96 (14.8%)	4 (8.0%)	1 (1.2%)	19 (15.3%)	0 (0.0%)	1 (2.9%)
Metal screw	2647 (13.7%)	2498 (13.6%)	92 (14.2%)	2 (4.0%)	31 (37.3%)	14 (11.3%)	3 (20.0%)	7 (20.0%)
Other	575 (3.0%)	540 (2.9%)	21 (3.2%)	3 (6.0%)*	6 (7.2%)***	2 (1.6%)	1 (6.7%)	2 (5.7%)
Meniscal injury (medial and/or lateral)								
Yes	8746 (45.0%)	8290 (44.8%)	302 (46.0%)	26 (50.0%)	50 (59.5%)**	57 (46.0%)	5 (33.3%)	16 (45.7%)
The Knee Injury and Osteoarthritis Outcome Score								
KOOS_4_ (preoperative)	52.1 (17.2) *n* = 12,645	52.3 (17.2) *n* = 12,063	49.3 (17.9)*** *n* = 425	45.3 (19.1)* *n* = 22	46.9 (17.0)* *n* = 49	50.9 (18.3) *n* = 66	35.7 *n* = 1	42.1 (15.4)* *n* = 19
Symptoms (preoperative)	67.5 (18.8) *n* = 12,643	67.6 (18.7) *n* = 12,061	64.9 (19.2)** *n* = 425	66.1 (24.5) *n* = 22	64.4 (18.9) *n* = 49	67.9 (20.6) *n* = 66	60.7 *n* = 1	63.9 (19.6) *n* = 19
Pain (preoperative)	72.0 (18.4) *n* = 12,644	72.1 (18.3) *n* = 12,062	70.4 (18.9) *n* = 425	67.7 (19.4) *n* = 22	68.5 (19.9) *n* = 49	70.7 (20.0) *n* = 66	58.3 *n* = 1	63.0 (21.4)* *n* = 19
Activities daily living (preoperative)	80.9 (18.2) *n* = 12,640	81.1 (18.1) *n* = 12,059	78.0 (19.4)*** *n* = 425	70.0 (23.1)* *n* = 21	75.2 (21.2) *n* = 49	78.9 (20.4) *n* = 66	80.9 *n* = 1	71.2 (20.1)* *n* = 19
Function sports and recreation (preoperative)	37.1 (26.7) *n* = 12,630	37.4 (26.6) *n* = 12,050	31.0 (26.6)*** *n* = 424	20.5 (24.9)** *n* = 21	27.0 (26.1)** *n* = 49	36.1 (29.8) *n* = 66	5.00 *n* = 1	19.2 (19.7)** *n* = 19
Quality of life (preoperative)	32.0 (17.7) *n* = 12,637	32.1 (17.6) *n* = 12,055	31.1 (20.0)* *n* = 425	27.8 (23.8) *n* = 22	27.8 (18.0) *n* = 49	28.8 (18.3) *n* = 66	18.8 *n* = 1	22.4 (13.4)* *n* = 19
Activity at time of injury								
Soccer	8536 (43.9%)	8248 (44.6%)	186 (28.3%)	11 (21.2%)	23 (27.4%)	55 (44.4%)	1 (6.7%)	12 (34.3%)
Other	10,919 (56.1%)	10,240 (55.4%)	471 (71.7%)***	41 (78.8%)***	61 (72.6%)**	69 (55.6%)	14 (93.3%)**	23 (65.7%)

For categorical variables number (*n*) and percentage (%) are presented. For continuous variables, the mean and standard deviation (SD) are presented

For comparisons between groups, Fisher’s exact test (lowest one-sided p-value multiplied by two) was used for dichotomous variables, the Chi square test was used for non-ordered categorical variables and the Mann–Whitney *U* test was used for continuous variables. The isolated ACL reconstruction group was used as reference and all the other study groups were compared with the isolated ACL group

*p < 0.05, **p < 0.01, ***p < 0.001

### Medial collateral ligament injury

#### Risk of ACL revision

The 2- and 5-year outcomes after isolated ACL reconstruction and combined ACL and MCL injury are presented in Table 2. There were no differences in the crude 2- and 5-year ACL revision rate between the groups. Factors that differed significantly in the pairwise comparison of each MCL group and the isolated ACL group and, in addition, were shown to correlate with both the predictor and the outcome were age, concomitant cartilage injury and the femoral fixation method. As a result, the Cox regression model was adjusted for age, patient sex, graft diameter, concomitant cartilage injury and femoral fixation method. Patients with an isolated ACL injury had a reduced risk of ACL revision compared with patients with a concomitant MCL injury (independent of type of treatment) (HR = 0.68 [95% CI 0.47–0.98], *p* = 0.036). When stratifying the analysis according to type of MCL treatment, the isolated ACL group had a significantly reduced risk of ACL revision compared with patients treated non-surgically for their MCL injury in both the unadjusted (HR = 0.69 [95% CI 0.48–0.99], *p* = 0.045) and adjusted analysis (HR = 0.61 [95% CI 0.41–0.89], *p* = 0.0097) but not compared with patients treated with either MCL suture repair or MCL reconstruction (Table 3). The planned analysis of the risk of revision between the different types of MCL treatment was not possible due to small study groups and few events in each group.

**Table 2:** Two- and 5-year outcomes for medial collateral ligament injury groups and isolated anterior cruciate ligament group

	Total (*n* = 19,283)	Isolated ACL (*n* = 18,490)	ACL + non-surgically treated MCL (*n* = 657)	ACL + MCL suture repair (*n* = 52)	ACL + MCL reconstruction (*n* = 84)	*p* value
ACL revision within 2 years						
Yes	368 (1.9%)	349 (1.9%)	19 (2.9%)	0 (0.0%)	0 (0.0%)	n.s
Contralateral ACL reconstruction within 2 years						
Yes	138 (0.7%)	133 (0.7%)	5 (0.8%)	0 (0.0%)	0 (0.0%)	
ACL revision within 5 years						
Yes	615 (3.2%)	586 (3.2%)	26 (4.0%)	2 (3.8%)	1 (1.2%)	n.s
Contralateral ACL reconstruction within 5 years						
Yes	382 (2.0%)	370 (2.0%)	11 (1.7%)	0 (0.0%)	1 (1.2%)	
MCL surgery with revision of ACL index operation						
Yes	11 (1.6%)	8 (1.2%)	2 (6.7%)	1 (50.0%)	0 (0.0%)	

For categorical variables, *n* (%) is presented. *n* = number. For comparisons between groups, the Chi square test was used

*ACL* anterior cruciate ligament, *MCL* medial collateral ligament, *n.s* non-significant

**Table 3:** Cox regression analysis for the risk of anterior cruciate ligament revision for medial collateral ligament injury

Groups compared	Hazard ratio unadjusted	*p* value	Hazard ratio adjusted*	*p* value adjusted*
Isolated ACL vs concomitant MCL (any treatment)	0.76 (0.53–1.07)	n.s	0.68 (0.47–0.98)	0.036
Isolated ACL vs ACL + non-surgically treated MCL	0.69 (0.48–0.99)	0.045	0.61 (0.41–0.89)	0.0097
Isolated ACL vs ACL + MCL suture repair	0.77 (0.19–3.09)	n.s	1.08 (0.15–7.67)	n.s
Isolated ACL vs ACL + MCL reconstruction	2.79 (0.39–19.82)	n.s	2.22 (0.31–15.82)	n.s

*ACL* anterior cruciate ligament, *MCL* medial collateral ligament, *n.s* non-significant

*Adjusted for age, patient sex, graft diameter, concomitant cartilage injury and femoral fixation method

#### Two-year patient-reported knee function

The non-surgically treated MCL group reported significantly lower 2-year KOOS in symptoms, sports and recreation and QoL compared with the isolated ACL group, with the sports and recreation subscale showing the largest mean difference (5.4 points [95% CI 1.8–9.0], *p* = 0.003). The MCL suture repair group reported significantly inferior scores in the similar subscales, with the largest mean difference in the sports and recreation subscale (13.1 points [95% CI 1.4–24.9], *p* = 0.028). The MCL reconstruction group reported significantly lower KOOS in symptoms (mean difference 8.0 points [95% CI 1.3–14.6], *p* = 0.018) and sports and recreation (mean difference 14.1 points [95% CI 4.3–23.9], *p* = 0.005) compared with the isolated ACL group. All MCL groups reported significantly inferior KOOS_4_ compared with the isolated ACL group, the MCL suture repair group showed the largest difference (9.8 points [95% CI 1.3–18.2], *p* = 0.024) (Table 4). Additional analyses comparing the 2-year KOOS between MCL suture repair and MCL reconstruction revealed no significant differences in any subscale, or in the KOOS_4_ (data not shown).

**Table 4:** Adjusted analysis for 2-year Knee Injury and Osteoarthritis Outcome Score between the isolated anterior cruciate ligament reconstruction and concomitant medial collateral ligament injury groups

Two-year KOOS domain	Isolated ACL^#^ (*n* = 6420)	ACL + non-surgically treated MCL (*n* = 236)			ACL + MCL suture repair (*n* = 21)			ACL + MCL reconstruction (*n* = 30)		
	Adjusted means* (95% CI)	Adjusted means* (95% CI)	Difference between groups adjusted means (95% CI)	Adjusted *p* value*	Adjusted means* (95% CI)	Difference between groups adjusted means (95% CI)	Adjusted *p* value*	Adjusted means* (95% CI)	Difference between groups adjusted means (95% CI)	Adjusted *p* value*
KOOS_4_	70.7 (70.2–71.2)	67.0 (64.4–69.5)	3.7 (1.2; 6.3)	0.005	60.9 (52.5–69.4)	9.8 (1.3; 18.2)	0.024	62.8 (55.7–69.8)	7.9 (0.8; 15.0)	0.028
Symptoms	76.6 (76.1–77.0)	73.5 (71.1–75.8)	3.1 (0.7; 5.5)	0.011	66.4 (58.5–74.3)	10.2 (2.3; 18.0)	0.012	68.6 (62.0–75.2)	8.0 (1.3; 14.6)	0.018
Pain	83.4 (83.0-83.8)	81.3 (79.1–83.4)	2.1 (− 0.1; 4.3)	n.s	78.2 (71.0–85.3)	5.2 (− 1.9; 12.4)	n.s	80.1 (74.2–86.1)	3.3 (− 2.7; 9.2)	n.s
ADL	90.3 (89.9–90.7)	89.6 (87.7–91.4)	0.7 (− 1.1; 2.6)	n.s	90.1 (83.9–96.2)	0.2 (− 5.9; 6.4)	n.s	88.4 (83.2–93.6)	1.9 (− 3.3; 7.1)	n.s
Sports and recreation	63.5 (62.9–64.2)	58.1 (54.6–61.6)	5.4 (1.8; 9.0)	0.003	50.4 (38.7–62.2)	13.1 (1.4; 24.9)	0.028	49.5 (39.7–59.3)	14.1 (4.3; 23.9)	0.005
QoL	59.3 (58.7–59.9)	55.0 (51.9–58.0)	4.3 (1.2; 7.4)	0.007	48.7 (38.5–59.0)	10.5 (0.2; 20.8)	0.045	52.9 (44.3–61.4)	6.4 (− 2.2; 15.0)	n.s

*ACL* anterior cruciate ligament, *ADL* activities of daily living, *CI* confidence interval, *MCL* medial collateral ligament, *n* number of patients, *n.s* non-significant, *QoL* quality of life

^#^The isolated ACL group was used as a reference group for all comparisons between groups

*Adjusting for age at surgery, patient sex and cartilage injury using analysis of covariance (ANCOVA)

### Lateral collateral ligament injury

#### Risk of ACL revision

The 2- and 5-year outcomes after isolated ACL reconstruction and combined ACL and LCL injury are presented in Table 5. There were no differences in the crude 2- and 5-year ACL revision rate between the groups. The prevalence of concomitant cartilage injury differed significantly in the pairwise comparison of each LCL group and the isolated ACL group and was additionally shown to correlate with both the predictor and the outcome. As a result, the Cox regression model was adjusted for patient sex, graft diameter and concomitant cartilage injury. The risk of ACL revision did not differ between patients with an isolated ACL reconstruction and any of the LCL treatment groups (Table 6). The planned analysis of the risk of ACL revision between the different types of LCL treatment was not possible to perform due to small study groups and few events in each group.

**Table 5:** Two- and five-year outcomes for lateral collateral ligament injury groups and isolated anterior cruciate ligament group

	Total (*n* = 18,664)	Isolated ACL (*n* = 18,490)	ACL + non-surgically treated LCL (*n* = 124)	ACL + LCL suture repair (*n* = 15)	ACL + LCL reconstruction (*n* = 35)	*p* value
ACL revision within 2 years						
Yes	351 (1.9%)	349 (1.9%)	1 (0.8%)	1 (6.7%)	0 (0.0%)	n.s
Contralateral ACL reconstruction within 2 years						
Yes	133 (0.7%)	133 (0.7%)	0 (0.0%)	0 (0.0%)	0 (0.0%)	
ACL revision within 5 years						
Yes	591 (3.2%)	586 (3.2%)	4 (3.3%)	1 (6.7%)	0 (0.0%)	n.s
Contralateral ACL reconstruction within 5 years						
Yes	371 (2.0%)	370 (2.0%)	1 (0.8%)	0 (0.0%)	0 (0.0%)	
LCL surgery with revision of ACL index operation						
Yes	11 (1.7%)	9 (1.4%)	1 (25.0%)	1 (100.0%)	0 (0.0%)	

For categorical variables, n (%) is presented. *n* = number. For comparisons between groups, the Chi square test was used

*ACL* anterior cruciate ligament, *LCL* lateral collateral ligament, *n* number of patients, n.s; non-significant

**Table 6:** Cox regression analysis for the risk of anterior cruciate ligament revision for lateral collateral ligament injury

Groups compared	Hazard ratio unadjusted	*p* value	Hazard ratio adjusted*	*p* value adjusted*
Isolated ACL vs concomitant LCL (any treatment)	1.18 (0.49–2.84)	n.s	1.11 (0.46–2.67)	n.s
Isolated ACL vs ACL + non-surgically-treated LCL	1.04 (0.39–2.78)	n.s	1.00 (0.37–2.67)	n.s
Isolated ACL vs ACL + LCL suture repair	0.60 (0.08–4.29)	n.s	0.56 (0.08–4.01)	n.s
Isolated ACL vs ACL + LCL reconstruction	22088.8 (0.00–^◊^)	n.s	18914.6 (0.00–^◊^)	n.s

*ACL* anterior cruciate ligament, *LCL* lateral collateral ligament, *n.s* non-significant

*Adjusted for patient sex, graft diameter and concomitant cartilage injury

^◊^No events in one of the groups means that the hazard ratio cannot be estimated

#### Two-year patient-reported knee function

The analysis of the 2-year KOOS was not possible to perform for the LCL suture repair and the LCL reconstruction groups, since 2-year data were only available for three and twelve patients, respectively, in these groups. A total of 46 patients within the non-surgically treated LCL group had available data for the 2-year KOOS. A non-surgically treated LCL injury resulted in significantly lower KOOS symptoms (mean difference 5.7 points [95% CI 0.4–11.0], *p* = 0.036), pain (mean difference 5.7 points [95% CI 0.8–10.5], *p* = 0.022) and sports and recreation (mean difference 9.2 points [95% CI 1.2–17.1], *p* = 0.024) compared with the isolated ACL group. The mean KOOS_4_ was 6.7 points (95% CI 1.0 –12.4, *p* = 0.022) lower in the non-surgically treated LCL group compared with the isolated ACL group (Table 7).

**Table 7:** Adjusted analysis for 2-year Knee Injury and Osteoarthritis Outcome Score between isolated anterior cruciate ligament reconstruction and concomitant non-surgically treated lateral collateral ligament injury

Two-year KOOS domain	Isolated ACL (*n* = 6420)	ACL + non-surgically treated LCL (*n* = 46)	Difference between groups adjusted means (95% CI)	Adjusted *p* value*
	Adjusted means* (95% CI)	Adjusted means* (95% CI)		
KOOS_4_	70.7 (70.2–71.2)	64.0 (58.3–69.7)	6.7 (1.0; 12.4)	0.022
Symptoms	76.6 (76.1–77.0)	70.8 (65.5–76.2)	5.7 (0.4; 11.0)	0.036
Pain	83.4 (83.0-83.8)	77.7 (72.9–82.5)	5.7 (0.8; 10.5)	0.022
ADL	90.3 (90.0-90.7)	87.0 (82.8–91.1)	3.4 (− 0.8; 7.5)	n.s
Sports and recreation	63.6 (62.9–64.2)	54.4 (46.5–62.3)	9.2 (1.2; 17.1)	0.024
QoL	59.3 (58.7–59.8)	52.9 (46.0–59.9)	6.3 (− 0.6; 13.3)	n.s

*ACL* anterior cruciate ligament, *ADL* activities of daily living, *CI* confidence interval, *LCL* lateral collateral ligament, *n* number of patients, *n.s* non-significant, *QoL* quality of life

*Adjusting for age at surgery, patient sex and cartilage injury using an analysis of covariance (ANCOVA)

## Discussion

The most important finding in this study was that the presence of a concomitant MCL injury was associated with an increased risk of undergoing ACL revision compared with an isolated ACL injury. The risk of ACL revision was significantly increased for patients undergoing ACL reconstruction and non-surgical treatment of their concomitant MCL injury, while patients treated with either MCL suture repair or reconstruction did not display an increased risk of ACL revision compared with isolated ACL reconstruction. This study also showed that the 2-year KOOS was significantly lower across all study groups in patients with a concomitant MCL injury compared with the isolated ACL injury group. However, patients undergoing surgical treatment for their MCL injury had a clinically relevant inferior KOOS compared with isolated ACL reconstruction, while the differences in the KOOS between the non-surgically treated MCL group and the isolated ACL group were small. The analyses of patients with a concomitant LCL injury were limited by small study groups, however, no significant difference in terms of the risk of ACL revision was found between patients with a concomitant LCL injury and patients with an isolated ACL injury.

The non-surgical treatment of isolated MCL injuries has been reported to produce a successful outcome, including high rates of return to sport [17, 25, 29]. Because of this, the controversy between existing studies of combined ACL and MCL injuries is mainly related to whether or not the MCL should be treated surgically. On one side, some authors have proposed that an early ACL reconstruction alone should be performed to minimize the gap between the torn ends of the MCL and accelerate healing without residual laxity [13, 14, 27]. Others have proposed that time should first be given, delaying surgery, to heal the MCL injury and that an isolated ACL reconstruction could be performed at a later stage, if severe valgus instability is not present intra-operatively [12]. The present study indicates that choosing non-surgical treatment of a concomitant MCL injury might increase the risk of necessitating a future ACL revision. One reason for this finding may be that laxity caused by MCL deficiency compromises the healing process of the newly reconstructed ACL. This theory is supported by biomechanical studies showing that MCL tears are associated with an increased load on the ACL [1, 43] and that forces not constrained by a deficient MCL will instead be mainly compensated for by the ACL [19]. As a result, the vulnerable ACL graft runs the risk of being subjected to excessive forces, which may predispose a subsequent graft failure.

The non-surgical treatment of a concomitant MCL injury has also been reported to increase the risk of residual laxity following ACL reconstruction compared with an isolated ACL reconstruction in the clinical setting [38]. If a severe medial-side injury is not properly diagnosed and treated accordingly, it may result in a complex laxity pattern of both valgus and anteromedial rotatory laxity [7]. The presence of this kind of residual laxity could contribute to a higher risk of ACL revision among non-surgically treated MCL injuries [1, 34, 35], which was shown in the present study. Nevertheless, the clinical decision-making regarding the choice of treatment for an MCL injury could be challenging and objective quantification of medial-side gapping by stress radiography could augment diagnostic power in addition to physical examination. A systematic review has, however, reported that valgus stress radiography has been underinvestigated in the literature in terms of diagnostic gapping benchmarks, accuracy and reliability [20]. Although there is a paucity of clinical studies on this subject, increased medial compartment gapping of 3.2 mm or more compared with the uninjured knee was shown to be indicative of a Grade III MCL tear in a cadaveric model [23]. The same study reported that gapping of more than 6.5 mm in full knee extension compared with the uninjured knee was indicative of an even more severe medial-side injury, likely also to involve other structures in the posteromedial corner [23]. There is no evidence that the spontaneous healing seen in superficial MCL injuries [36] could be expected for more extensive injuries to the medial structures, which underscores the importance of identifying MCL injuries requiring surgical treatment. However, future in vivo research regarding medial-side knee injuries is warranted to determine how the degree of medial gapping should be incorporated in the treatment algorithm.

Two factors are especially important to consider when using ACL revision as the primary endpoint in the SNKLR. First, there is a risk that graft failures and inferior outcomes for patients who choose not to undergo ACL revision will not be distinguished. Second, the SNKLR does not include information on return to sport or activity level, which could per se influence a patient’s risk of sustaining a new ACL injury requiring revision. It has previously been reported that the risk of ACL revision is highest during the first two postoperative years, likely due to the fact that most patients return to knee-strenuous activity at some time during this timeframe [22, 24]. This study found that no ACL revision was registered during the first two  years in either the MCL repair or the reconstruction group. Does this mean that these patients had such severe knee problems that they were not exposed to the risk of re-injury while participating in knee-strenuous activity, which might thereby explain the lower risk of ACL revision? Interestingly, a recent study showed that a concomitant MCL injury was a strong negative predictor of return to knee-strenuous sport within the first postoperative year after ACL reconstruction [15]. Moreover, the MCL repair and the MCL reconstruction groups in present study reported almost 15 points lower in the sports and recreation subscale compared with the isolated ACL group, while the non-surgically treated MCL group reported only a slightly inferior KOOS. This finding suggests that patients in the MCL repair and reconstruction groups might adapt their activity demands and thereby run a lower risk of sustaining a new ACL injury requiring revision, thereby underscoring the limitation of using the event of ACL revision as an outcome in the present study.

The analyses of patients with an LCL injury were limited by small study groups, which means that these findings should be interpreted carefully. There was no difference in the risk of ACL revision between an isolated ACL injury and any type of treatment for an LCL injury. However, only four patients in the non-surgically treated LCL group and one patient in the LCL repair group underwent ACL revision during the first five postoperative years, which are small numbers to analyze. Moreover, no clinically important differences were found between the non-surgically treated LCL group and the isolated ACL group in the 2-year KOOS.

The unspecified grade of the concomitant collateral ligament injuries in the present study limits the ability to draw conclusions. It must also be stated that the study does not differentiate between the degree of valgus or varus laxity among the investigated patients. Nor is the surgical technique for the ACL reconstruction or the collateral ligament procedures known and the treatment was performed by several surgeons. Moreover, there is no information about the pre- or postoperative rehabilitation or the activity level among included patients. Nonetheless, this is the first study from the SNKLR to investigate the impact of collateral ligament injury on the risk of ACL revision, which permits the investigation of one of the largest population-based cohorts for combined ACL and collateral ligament injuries to date. The findings of this study should be considered as one component in the clinical decision-making regarding management of combined ACL and collateral ligament injuries, indicating that non-surgical treatment of the latter potentially increases the risk of ACL revision.

## Conclusion

Non-surgical treatment of a concomitant MCL injury in the setting of an ACL reconstruction may increase the risk of ACL revision. Clinicians should thoroughly consider the need for surgical treatment for a concomitant MCL injury to prevent residual laxity, potentially increasing the risk of ACL revision. However, surgical treatment of the MCL injury was associated with worse two-year patient-reported knee function. A concomitant LCL injury does not impact the risk of ACL revision compared with an isolated ACL reconstruction.

## References

[1] Battaglia MJ, Lenhoff MW, Ehteshami JR, Lyman S, Provencher MT (2009). Medial collateral ligament injuries and subsequent load on the anterior cruciate ligament: a biomechanical evaluation in a cadaveric model. Am J Sports Med.

[2] Borden PS, Kantaras AT, Caborn DN (2002). Medial collateral ligament reconstruction with allograft using a double-bundle technique. Arthroscopy.

[3] Covey DC (2001). Injuries of the posterolateral corner of the knee. J Bone Jt Surg Am.

[4] Dong J, Wang XF, Men X, Zhu J, Walker GN (2015). Surgical treatment of acute grade III medial collateral ligament injury combined with anterior cruciate ligament injury: anatomic ligament repair versus triangular ligament reconstruction. Arthroscopy.

[5] Dong JT, Chen BC, Men XQ, Wang F, Hao JD (2012). Application of triangular vector to functionally reconstruct the medial collateral ligament with double-bundle allograft technique. Arthroscopy.

[6] Emilsson L, Lindahl B, Koster M, Lambe M, Ludvigsson JF (2015). Review of 103 Swedish Healthcare Quality Registries. J Intern Med.

[7] Engebretsen L, Lind M (2015). Anteromedial rotatory laxity. Knee Surg Sports Traumatol Arthrosc.

[8] Frobell RB, Roos EM, Roos HP, Ranstam J, Lohmander LS (2010). A randomized trial of treatment for acute anterior cruciate ligament tears. N Engl J Med.

[9] Gallo RA, Kozlansky G, Bonazza N, Warren RF (2017). Combined anterior cruciate ligament and medial collateral ligament reconstruction using a single achilles tendon allograft. Arthrosc Tech.

[10] Geeslin AG, Moulton SG, LaPrade RF (2016). A systematic review of the outcomes of posterolateral corner knee injuries, part 1: surgical treatment of acute injuries. Am J Sports Med.

[11] Gollehon DL, Torzilli PA, Warren RF (1987). The role of the posterolateral and cruciate ligaments in the stability of the human knee. A biomechanical study. J Bone Jt Surg Am.

[12] Grant JA, Tannenbaum E, Miller BS, Bedi A (2012). Treatment of combined complete tears of the anterior cruciate and medial collateral ligaments. Arthroscopy.

[13] Halinen J, Lindahl J, Hirvensalo E (2009). Range of motion and quadriceps muscle power after early surgical treatment of acute combined anterior cruciate and grade-III medial collateral ligament injuries. A prospective randomized study. J Bone Jt Surg Am.

[14] Halinen J, Lindahl J, Hirvensalo E, Santavirta S (2006). Operative and nonoperative treatments of medial collateral ligament rupture with early anterior cruciate ligament reconstruction: a prospective randomized study. Am J Sports Med.

[15] Hamrin Senorski E, Svantesson E, Beischer S, Thomee C, Thomee R (2018). Low 1-year return-to-sport rate after anterior cruciate ligament reconstruction regardless of patient and surgical factors: a prospective cohort study of 272 Patients. Am J Sports Med.

[16] Hillard-Sembell D, Daniel DM, Stone ML, Dobson BE, Fithian DC (1996). Combined injuries of the anterior cruciate and medial collateral ligaments of the knee. Effect of treatment on stability and function of the joint. J Bone Jt Surg Am.

[17] Holden DL, Eggert AW, Butler JE (1983). The nonoperative treatment of grade I and II medial collateral ligament injuries to the knee. Am J Sports Med.

[18] Indelicato PA (1983). Non-operative treatment of complete tears of the medial collateral ligament of the knee. J Bone Jt Surg Am.

[19] Inoue M, McGurk-Burleson E, Hollis JM, Woo SL (1987). Treatment of the medial collateral ligament injury. I: the importance of anterior cruciate ligament on the varus-valgus knee laxity. Am J Sports Med.

[20] James EW, Williams BT, LaPrade RF (2014). Stress radiography for the diagnosis of knee ligament injuries: a systematic review. Clin Orthop Relat Res.

[21] Kitamura N, Ogawa M, Kondo E, Kitayama S, Tohyama H (2013). A novel medial collateral ligament reconstruction procedure using semitendinosus tendon autograft in patients with multiligamentous knee injuries: clinical outcomes. Am J Sports Med.

[22] Kyritsis P, Bahr R, Landreau P, Miladi R, Witvrouw E (2016). Likelihood of ACL graft rupture: not meeting six clinical discharge criteria before return to sport is associated with a four times greater risk of rupture. Br J Sports Med.

[23] Laprade RF, Bernhardson AS, Griffith CJ, Macalena JA, Wijdicks CA (2010). Correlation of valgus stress radiographs with medial knee ligament injuries: an in vitro biomechanical study. Am J Sports Med.

[24] Lind M, Menhert F, Pedersen AB (2012). Incidence and outcome after revision anterior cruciate ligament reconstruction: results from the Danish registry for knee ligament reconstructions. Am J Sports Med.

[25] Lundberg M, Messner K (1996). Long-term prognosis of isolated partial medial collateral ligament ruptures. A ten-year clinical and radiographic evaluation of a prospectively observed group of patients. Am J Sports Med.

[26] Marchant MH, Tibor LM, Sekiya JK, Hardaker WT, Garrett WE (2011). Management of medial-sided knee injuries, part 1: medial collateral ligament. Am J Sports Med.

[27] Millett PJ, Pennock AT, Sterett WI, Steadman JR (2004). Early ACL reconstruction in combined ACL-MCL injuries. J Knee Surg.

[28] Petersen W, Laprell H (1999). Combined injuries of the medial collateral ligament and the anterior cruciate ligament. Early ACL reconstruction versus late ACL reconstruction. Arch Orthop Trauma Surg.

[29] Reider B, Sathy MR, Talkington J, Blyznak N, Kollias S (1994). Treatment of isolated medial collateral ligament injuries in athletes with early functional rehabilitation. A five-year follow-up study. Am J Sports Med.

[30] Robinson JR, Sanchez-Ballester J, Bull AM, Thomas Rde W, Amis AA (2004). The posteromedial corner revisited. An anatomical description of the passive restraining structures of the medial aspect of the human knee. J Bone Jt Surg Br.

[31] Roos EM, Roos HP, Lohmander LS, Ekdahl C, Beynnon BD (1998). Knee Injury and Osteoarthritis Outcome Score (KOOS)—development of a self-administered outcome measure. J Orthop Sports Phys Ther.

[32] Shelbourne KD, Carr DR (2003). Combined anterior and posterior cruciate and medial collateral ligament injury: nonsurgical and delayed surgical treatment. Instr Course Lect.

[33] Stannard JP, Brown SL, Farris RC, McGwin G, Volgas DA (2005). The posterolateral corner of the knee: repair versus reconstruction. Am J Sports Med.

[34] Wijdicks CA, Griffith CJ, Johansen S, Engebretsen L, LaPrade RF (2010). Injuries to the medial collateral ligament and associated medial structures of the knee. J Bone Jt Surg Am.

[35] Wijdicks CA, Griffith CJ, LaPrade RF, Spiridonov SI, Johansen S (2009). Medial knee injury: part 2, load sharing between the posterior oblique ligament and superficial medial collateral ligament. Am J Sports Med.

[36] Woo SL, Vogrin TM, Abramowitch SD (2000). Healing and repair of ligament injuries in the knee. J Am Acad Orthop Surg.

[37] Woo SL, Young EP, Ohland KJ, Marcin JP, Horibe S (1990). The effects of transection of the anterior cruciate ligament on healing of the medial collateral ligament. A biomechanical study of the knee in dogs. J Bone Jt Surg Am.

[38] Zaffagnini S, Bignozzi S, Martelli S, Lopomo N, Marcacci M (2007). Does ACL reconstruction restore knee stability in combined lesions?: an in vivo study. Clin Orthop Relat Res.

[39] Zaffagnini S, Bonanzinga T, Marcheggiani Muccioli GM, Giordano G, Bruni D (2011). Does chronic medial collateral ligament laxity influence the outcome of anterior cruciate ligament reconstruction?: a prospective evaluation with a minimum three-year follow-up. J Bone Jt Surg Br.

[40] Zantop T, Schumacher T, Diermann N, Schanz S, Raschke MJ (2007). Anterolateral rotational knee instability: role of posterolateral structures. Winner of the AGA-DonJoy Award 2006. Arch Orthop Trauma Surg.

[41] Zantop T, Schumacher T, Schanz S, Raschke MJ, Petersen W (2010). Double-bundle reconstruction cannot restore intact knee kinematics in the ACL/LCL-deficient knee. Arch Orthop Trauma Surg.

[42] Zhang H, Sun Y, Han X, Wang Y, Wang L (2014). Simultaneous reconstruction of the anterior cruciate ligament and medial collateral ligament in patients with chronic ACL-MCL lesions: a minimum 2-year follow-up study. Am J Sports Med.

[43] Zhu J, Dong J, Marshall B, Linde MA, Smolinski P (2018). Medial collateral ligament reconstruction is necessary to restore anterior stability with anterior cruciate and medial collateral ligament injury. Knee Surg Sports Traumatol Arthrosc.

